# Dormancy of cutaneous melanoma

**DOI:** 10.1186/s12935-024-03278-5

**Published:** 2024-02-28

**Authors:** Kathrin Singvogel, Birgit Schittek

**Affiliations:** 1 Division of Dermatooncology, Department of Dermatology, University of Tübingen, Liebermeisterstr. 25, D -72076 , Tübingen, Germany; 2 Cluster of Excellence iFIT (EXC 2180) “Image-Guided and Functionally Instructed Tumor Therapies”, University of Tübingen, Tübingen, Germany

**Keywords:** Melanoma, Dormant cancer, Neutrophils, NETs, Immunotherapy

## Abstract

Many cancer-related deaths including melanoma result from metastases that develop months or years after the initial cancer therapy. Even the most effective drugs and immune therapies rarely eradicate all tumor cells. Instead, they strongly reduce cancer burden, permitting dormant cancer cells to persist in niches, where they establish a cellular homeostasis with their host without causing clinical symptoms. Dormant cancers respond poorly to most drugs and therapies since they do not proliferate and hide in niches. It therefore remains a major challenge to develop novel therapies for dormant cancers. In this review we focus on the mechanisms regulating the initiation of cutaneous melanoma dormancy as well as those which are involved in reawakening of dormant cutaneous melanoma cells. In recent years the role of neutrophils and niche components in reawakening of melanoma cells came into focus and indicate possible future therapeutic applications. Sophisticated in vitro and in vivo melanoma dormancy models are needed to make progress in this field and are discussed.

## Introduction

Advanced malignant melanoma still has a poor prognosis. Melanoma originates from malignant transformation of melanocytes in the skin, uvea, or mucosa. Around 23,000 new cases and around 3,000 deaths per year are reported in Germany [1] with a 2.5–6% annual increase in incidence since the 1990s [2]. Melanoma can be divided into four stages which are defined by thickness, ulceration, and metastasis. Stages I and II are still localized and likely to have a good prognosis, whereas stages III and IV are defined by regional (III) and distant (IV) metastasis [3]. For stage IV melanoma, the prognosis remains poor, with a five year survival rate of approximately 25% in Germany [1]. Although surgical approaches combined with new therapeutic strategies, such as immunotherapy, significantly prolong recurrence-free survival of stage III melanoma, > 50% of patients relapse after 3 years [4]. Interestingly, relapse of stage I melanoma patients may be delayed for years or even decades [5]. Recurrence occurs locally (19.6%), in regional lymph nodes (29.8%) or at distant sites (50.6%), such as lung, brain and in intraabdominal or bone locations [6]. The underlying mechanism of late recurrence is thought to be tumor dormancy. Tumor dormancy and metastatic recurrence are nowadays a significant diagnostic and therapeutic challenge, the mechanism for which must first be understood [7]. Many of these mechanisms have been primarily discovered in other tumor types than melanoma and will therefore also be discussed here.

## Hallmarks of tumor dormancy

Cancer dormancy encompasses two distinct states, which are not mutually exclusive: tumor mass dormancy on a population-level and cellular dormancy [8]. Tumor mass dormancy is characterized by a balance of tumor-cell proliferation and death keeping the tumor cell population at a constant level. Cellular dormancy describes a state of growth arrest on a single cell level in which cells have undergone a reversible G0-G1 cell cycle arrest. Dormant cancer cells are defined by three essential properties: they persist within a foreign microenvironment, they are reversibly growth-arrested and they resist targeted and cytotoxic treatments. It is increasingly appreciated that the microenvironment has an important role in conferring and maintaining these states [8].

Cancer dormancy is under dynamic control and there are a series of stages in the life cycle of a dormant cancer cell that include niche dependence, cell cycle arrest, drug resistance, immune evasion, metastatic relapse, and reversibility [9]. As a first step, the disseminated cancer cells (DCCs) have to find the right supportive niches and occupy those. It is well known that tumor cells have a preference for specific organs to home, e.g. melanoma cells prefer lymph nodes, bone, lung, liver, skin and brain [10, 11]. Second, DCCs have to interact with the niche components as Steven Paget proposed already over 100 years ago in the “seed and soil” hypothesis [12]. It is also now well established that cancer cells prepare a metastatic niche (so called pre-metastatic niche) before leaving the primary tumor [13]. Third, the DCCs have to adapt to the niche by cellular reprogramming to establish a reversible growth-arrested state and to evade immunosurveillance. Many evidences until now clearly show that dormancy is not solely a cell-intrinsic property and that the niche is actively involved in the reprogramming of tumor cells to keep them either dormant or to reactivate them [9, 14]. In particular, the interaction of distinct immune cells with the tumor microenvironment resulting in remodeling of the niche and in reactivation of dormant cancer cells is of importance [9]. Clearly, a more detailed understanding of the interplay of dormant cancer cells and the niche components is needed to understand the mechanism of dormancy induction, maintenance and escape.

Dormant cancer cells share some characteristics with other cells undergoing proliferative arrest, namely senescent cells and quiescent cancer stem cells [15]. “Senescence at the cellular level” was described as early as 1961 by Hayflick and Moorhead, who found a limited ability of diploid cells to divide [16]. It is generally understood to be an irreversible cell cycle arrest with altered phenotype [17, 18]. Nevertheless, re-entry into the cell cycle was proven to be possible under certain circumstances in an in vitro model of lymphoma cells [19]. In addition to cell cycle arrest, the senescent associated secretory phenotype (SASP), macromolecular damage, and altered metabolism are counted as hallmarks of senescence [18].

Senescence cannot be defined by a specific marker, but only by the synopsis of senescence-associated characteristics [18]. Cytoplasmic markers such as β-galactosidase, as well as lipofuscin detection with Sudan-Black-B stain, detect metabolic, lysosomal changes [20, 21]. Cell cycle arrest is often induced by p16 and p21, which inhibit cyclin-dependent kinases and, thus, preventing cell cycle progression [22, 23]. At the same time, proliferation markers, such as Ki-67, are negative [24]. In addition, a highly variable spectrum of SASP components, such as the proinflammatory cytokines IL-6 and IL-8, are secreted [25]. Senescence is a response to stressors, which include, for example, activation of oncogenes, telomere shortening or genotoxic substances [26–28]. In contrast to senescence, tumor dormancy is a clinical term that describes remaining growth-arrested cells in the patient. However, since senescent and dormant cells exhibit overlapping characteristics, a continuum between both states may be assumed [29].

Besides a cellular and a tumor mass dormancy, the existence of cancer stem cells, often referred to as “slow-cycling cells”, has been described [9, 30]. They differ from the dormant tumor cell by low differentiation and stem cell markers and no definitive growth arrest [31–33]. A hierarchical organization with few tumor stem cells has been described for colorectal cancer [32]. The model is challenged by a xenotransplantation model in melanoma in which 25% of unselected melanoma cells were able to form tumors under permissive conditions [34].

## Clinical evidence of melanoma dormancy

Cancer dormancy was originally defined by Willis in the late 1940s and later by Hadfield in the early 1950s as a temporary growth arrest [35, 36]. Cells can detach from a primary tumor and travel as DCCs through blood vessels to new sites in the body. After settling into other tissues, they initially hide in a quiescent state in their niche, where they are protected from the immune system or actively suppress immune responses [37, 38]. DCCs are already found in the lymph node very early after primary melanoma development [39] and the number of DCCs in the sentinel and regional lymph nodes correlates with the prognosis of melanoma patients [40, 41]. Their clinical and mouse model data suggest a parallel progression with early dissemination of melanoma cells, as 50% of tumor spread occurs in tumors of < 0.5 mm thickness [39]. As further proven by Han et al., DCCs of melanoma and breast cancer were already detected at a pre-metastatic stage in the lymphatic vessels of a mouse model [42]. Early DCCs may prepare the tumor niche into which late DCCs may settle [43]. Clinical existence of dormant melanoma cells is underlined by an impressive example: various recipients of kidney transplants from immune competent donors, who had a primary melanoma removed up to 32 years prior to the organ donation, developed melanoma metastases in the immune suppressed recipients that were derived from dormant tumor cells in the kidney grafts [44]. There exist several other examples of the inadvertent transmission of dormant melanoma cells from a donor to the organ transplant recipient [45] or of recurrence more than 10 years after diagnosis of stage I-III melanoma [46–49]. All of these data indicates that under specific conditions, especially under impaired host immunity, dormant tumor cells awake, acquire proliferative capacity and disseminate into other target organs. The trigger factors involved in reawakening of dormant tumor cells are still not identified, but an active immune response is likely to be involved.

## Dissemination of melanoma cells and metastasis

**Fig. 1:**
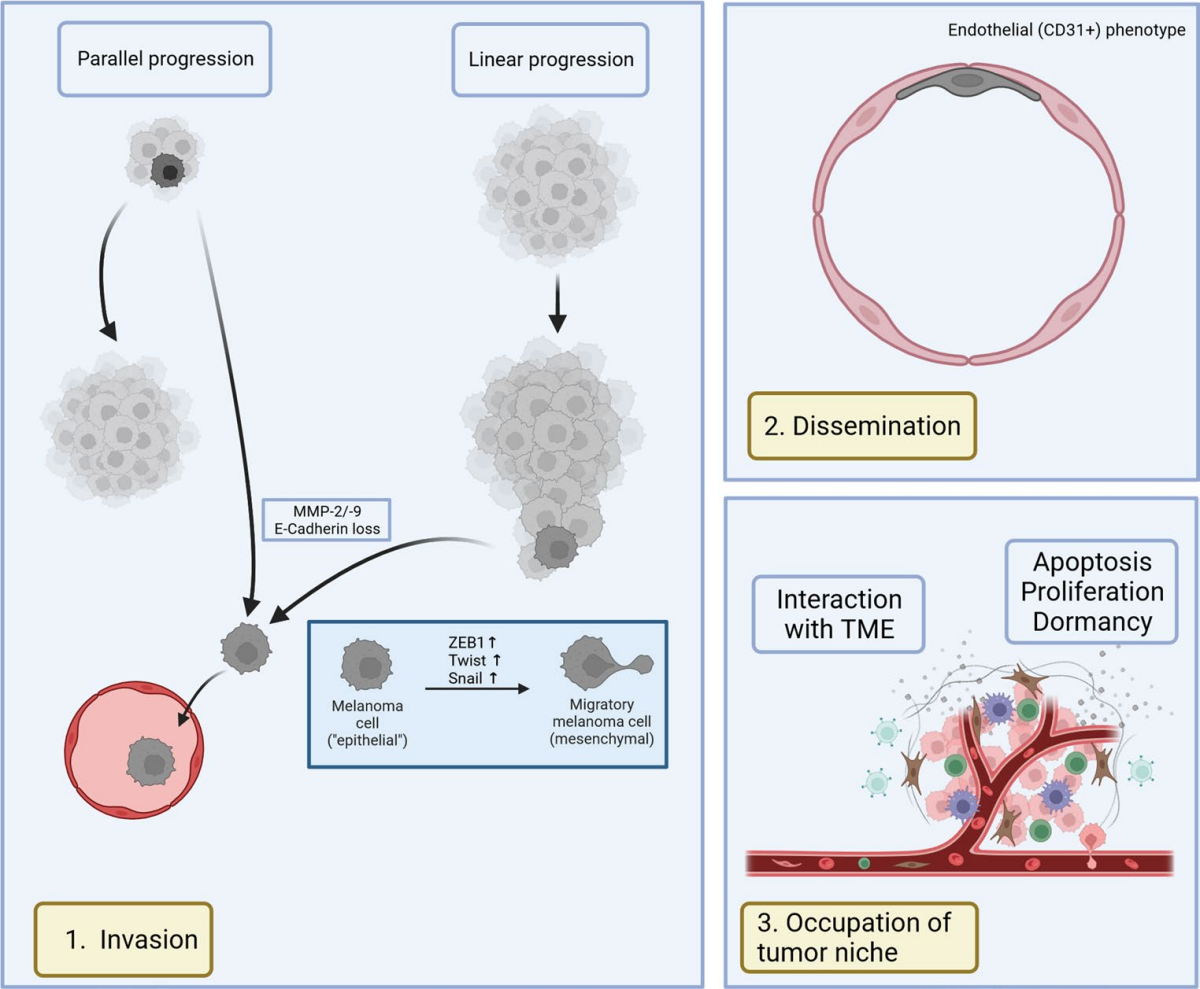
Metastatic process of melanoma. **1**. Melanoma cells may detach from the tumor in parallel to the growth of the primary tumor or in a linear process in which tumors first accumulate genetic and epigenetic alterations after which tumor cells disseminate. They undergo an epithelial-mesenchymal-like transition for higher invasive potential before entering the lymphatic or vascular system. **2**. Melanoma cells circulate within the body and may adapt an endothelial phenotype within the vascular niche. **3**. Melanoma cells occupy their niche and interact with the tumor microenvironment (TME) which includes extracellular matrix (ECM), cellular components and cytokines. They may undergo apoptosis, or either enter a proliferative or dormant state (created with BioRender.com)

Metastatic progression is assumed to be either linear or parallel [43, 50]. In 1989, Clark et al. describes metastasis as a linear process in which tumors accumulate genetic and epigenetic alterations and progress from radial, to vertical, to invasive growth phases after which tumor cells disseminate [51]. In the linear model one expects a high degree of similarity between the primary tumor, the DCCs and its metastases [50]. However, tumor cell dissemination can also occur early when the primary tumor is still very small and progress at the metastatic site in parallel to the progression of the primary tumor which might continue to seed cells. In the parallel model one expects a higher degree of genomic diversity between the metastases and the primary tumor [52] (Fig. 1).

Independent of a linear or parallel progression pathway disseminated melanoma cells must first gain access to the lymphatic system, through which they preferentially disseminate (Fig. 1). To clear a pathway, melanoma cells secrete the metalloproteinases MMP-2 and MMP-9 that degrade the extracellular matrix (ECM), or they stimulate surrounding fibroblasts to release MMP-1 under in vitro and in vivo conditions [53–55]. Loss of adhesion molecules such as E-cadherin interrupts intercellular junctions between melanoma cells and keratinocytes, therefore allowing migration [56]. E-Cadherin repression in a cell culture melanoma model is regulated by epithelial-mesenchymal transition (EMT) promoting transcription factors such as Slug, Zinc Finger E-Box Binding Homeobox 1 (ZEB1), Twist and Snail [56, 57]. EMT marker expression in patient samples is associated with a metastatic, invasive phenotype in melanoma [58]. Another delineating feature of disseminated melanoma cells in patients is their lower BRAF mutation rate, making them distinct from the proliferating primary tumor cells [39].

In the second step, tumor cells spread throughout the body via the lymphatic and blood systems [42]. Melanoma cells may remain in an intravascular niche within secondary organs, such as the lung. In vivo lineage tracing in a mouse model revealed a switch to an endothelial quiescent phenotype (CD31+) with loss of typical melanoma markers, emphasizing the plasticity of melanoma cells [59] (Fig. 1).

Thirdly, DCCs actively transmigrate through the vascular wall to occupy the tumor niche. Prior to occupancy, primary tumors including melanoma may shed soluble factors, such as exosomes, to prepare a favorable “soil”, the pre-metastatic niche in mice and humans [13, 60]. After settlement, DCCs interact with components of the microenvironment, which consists of ECM, cells (fibroblasts, immune cells) and soluble factors (cytokines, chemokines). The further fate of the melanoma cell depends on cell-intrinsic properties (“seed”) and its environment (“soil”) [12] (Fig. 1). As demonstrated by Eyles et al. in RET.AAD mice, few of the disseminated cells undergo apoptosis. Remaining melanoma cells maintain a dormant state in the presence of CD8 + T cells while proliferating in the absence of immune control [61].

## Molecular and cellular mechanisms regulating melanoma dormancy

### Melanoma plasticity and phenotype switching

The life cycle of a cancer cell – including transitions from circulating DCC to the growth-arrested cancer cell in the distinct niches up to the escape from the dormant state - requires a high plasticity of the cells, which is influenced by niche components [62]. As DCCs may contain fewer genetic aberrations compared to primary or metastatic tumors in the same patient, dissemination of melanoma and breast cancer cells is often an early event during cancer progression and epigenetic changes allow adaption to new circumstances [39, 63–65].

Melanoma cells can reversibly switch between a proliferative and an invasive phenotype, a model which is called the phenotype switching model first described by Hoek et al. [66, 67]. Both cell states can co-exist in the tumors of patients emphasizing the intratumor heterogeneity [68, 69]. Interestingly, temporal single-cell tracing in mice indicates that only the population of melanoma cells with an invasive, mesenchymal-like state constitute a pool of metastatic initiating cells that switch cell identity while disseminating to secondary organs [68]. Phenotype-switching is described to be mainly based on the relative expression of the transcription factors microphtalmia transcription factor (MITF) and SOX10. Whereas cells expressing low levels of MITF have a slow-cycling, mesenchymal-like, pro-invasive phenotype, higher levels of MITF correlate with proliferation and differentiation [69–72]. Interestingly, low MITF levels are also found in drug resistant melanoma cell lines, which was previously defined as hallmark of dormancy [73]. MITF acts as a “rheostat” and is considered as one of the key regulators of melanoma phenotype switching [71, 74]. SOX10 can promote MITF expression and SOX10 downregulation induces a slow proliferating mesenchymal-like state [75, 76]. SOX10 depletion in melanoma cells in vitro induces an invasive-like state and a dormant-like phenotype in vivo [77]. Intriguingly, loss of SOX10 leads to SOX9 upregulation in an antagonistic fashion [78]. In melanoma, SOX9 is a driver of the mesenchymal-like state [79]. Epithelial to mesenchymal transition (EMT)-like processes enable melanoma cells to disseminate from the primary site [76, 80] and this is orchestrated by the main transcription factors Snail, Slug, ZEB1 and Twist1, whereas their repression is required to promote metastatic growth in vivo [81, 82].

Dormant cancer cells have distinct transcriptional and epigenetic profiles compared with proliferating cancer cells and few evidences yet indicate that they may have slowed their metabolic rate, are more dependent on glucose, glutamine and fatty acid metabolism and have been shown to have increased mitochondrial activity [9, 83, 84]. However, there is no universally valid marker for dormant melanoma cells and, therefore, no uniform detection method. In melanoma the transforming growth factor β (TGF-β) is linked to dormancy since it promotes cell migration, immune escape, and metastasis [85, 86]. Blocking TGF-β can prevent the development of melanoma bone metastases and decrease the progression of established osteolytic lesions [87]. On molecular level, blocking of TGF-β in a melanoma in vitro model results in a GLI2^low^/MITF^high^ phenotype, which in turn leads to reduced WNT5A expression and to less invasive capacities [88].

Besides cell-intrinsic factors regulating proliferation, migration and invasion, micro-environmental factors such as nutrients, oxygen, cytokines, and growth factors influence the reversible switch [62, 76]. Hypoxia and inflammatory signals from the microenvironment are able to promote the switch from a proliferative to an invasive phenotype [89–92]. Inflammation of the lung can induce EMT of dormant breast cancer cells in a mouse model via the expression of ZEB1, resulting in reactivation of the dormant cancer cells [93].

There is a constant crosstalk of the dormant cancer cells with cells in the niche to modulate ECM components to either keep the cells dormant or getting them reactivated and inducing metastatic spread [94, 95]. Dormant breast cancer cells can activate stromal cells to release niche ECM components such as periostin and tenascin C, which in turn activate tumorigenic stem cell signaling pathways such as Wnt, Nanog and Oct4 in the cancer cells leading to their metastatic outgrowth [80]. Tissue stiffness and matrix composition are important parameters in the induction of dormancy in melanoma cells in vitro [96, 97]. Matrix metalloproteinases (MMPs), which can degrade ECM-components are associated with melanoma progression [98]. Extracellular vesicles secreted by melanoma cells can reshape the premetastatic niche facilitating colonization of disseminated melanoma cells [13, 60, 99].

#### Signaling pathways

Melanoma cells have a high degree of plasticity [62]. Low levels of Ki-67, as well as high levels of cyclin-dependent kinase (CDK) inhibitor p27, are indicative of melanoma cell dormancy [100]. The MAPK signaling pathway plays a role in proliferation, growth, differentiation, migration, and apoptosis [101]. In breast cancer, and head and neck carcinoma, low ERK 1/2 activity with increased activation of p38 (ERK^low^/p38^high^) is indicative of dormancy [102, 103]. Consequently, cell cycle regulators, such as p27 and the orphan nuclear receptor NR2F1, are increasingly expressed, triggering growth arrest [104, 105]. In cell culture models of melanoma, ERK and p38 are phosphorylated simultaneously and promote proliferation, hence an inactivation of both proteins may be assumed during growth stagnation [106]. Interestingly, p38 modulates key players of the unfolded protein response involved in the ER stress pathways suggesting that dormant melanoma cells are in a state of cellular stress [97].

The PI3K/AKT signaling pathway is activated by receptor tyrosine kinases (RTK) and promotes growth and survival of melanoma cells [107–109]. When downstream targets such as PHF19 are silenced, the phenotype of melanoma cells in a spheroid model shifts from a proliferative to an invasive state [110]. Glucocorticoid-induced leucine zipper (GILZ) deactivates a downstream target of the PI3K/AKT pathway in a murine melanoma model, and its repression increases p21 levels to induce a cell cycle arrest [31].

The STAT3 signaling pathway opens the gateway towards proliferation or apoptosis in dormant melanoma cells. Induced by SOX2 depletion or interferon (IFN)β, activated P-STAT3 binds abundantly to the p53 promoter and enhances the apoptotic pathway in an in vitro melanoma model [111, 112]. Conversely, knockout of STAT3 increases MITF transcription and leads to a proliferative phenotype. Intermediate STAT3 activation with a MITF^low^ phenotype is expected to keep melanoma cells in the dormant state [113]. Further dormancy maintaining genes and modulating factors in melanoma are reviewed by Janowska et al. [114].

## Mechanisms of melanoma dormancy induction and reawakening

**Fig. 2:**
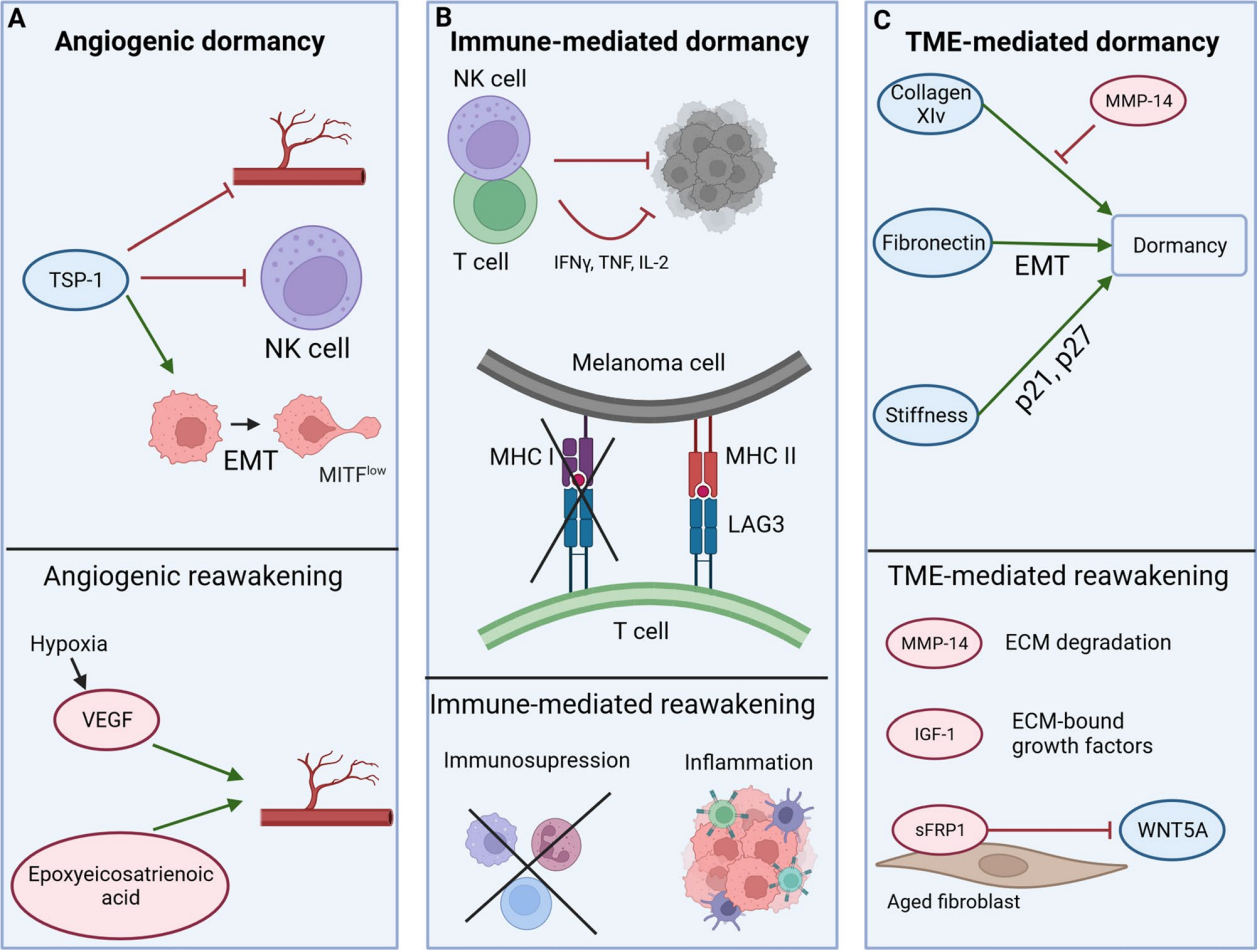
Mechanisms of dormancy induction. Dormancy can be classified into angiogenic (**A**), immune-mediated (**B**), and tumor microenvironment (TME)-mediated (**C**) dormancy and can be influenced by growth arresting and reawakening factors (created with BioRender.com). More information can be found in the text

Dormancy can be differentiated into angiogenic (A), immune-mediated (B), and tumor microenvironment (TME)-mediated (C) dormancy (Fig. 2). Growth arresting and reawakening factors can be assigned to these categories [29, 115].

Angiogenic dormancy refers to nutritional deprivation, as well as hypoxia in the absence of adequate vascularization, leading to an equilibrium between apoptosis and proliferation within the micrometastasis [116, 117]. A central role is attributed to the angiogenesis inhibitor thrombospondin-1 (TSP-1), which is secreted by immune cells or melanoma cells in a xenograft model [118, 119]. In addition to its anti-angiogenic effect, TSP-1 is involved in the induction of a mesenchymal invasive MITF^low^ phenotype (EMT-like process) of melanoma cells in vitro and embryonic chicken neural tube and is found to be aberrantly expressed in biopsies of metastatic melanoma [120]. TSP-1 further controls the suppression of natural killer (NK) cells in vitro, possibly blocking immune surveillance of melanoma in vivo (Fig. 2A) [121]. Expanding the definition of angiogenic dormancy is reasonable, as individual melanoma cells may remain dormant in the perivascular niche with abundant nutrient supply. Therefore, vascular-derived factors may prevent proliferation [97, 122]. Angiogenic dormancy may be disrupted in a process called the angiogenic switch [123]. In melanoma, proangiogenic factors, such as vascular endothelial growth factor (VEGF) and epoxyeicosatrienoic acid of the endothelium, allow vascularization in mice (Fig. 2A) [124, 125]. It should be noted that during hypoxia- an inducer of angiogenic dormancy- target genes of hypoxia-induced factors (HIF) such as VEGF are increasingly expressed in cultured melanoma cells, indicating a contrary role of hypoxia in dormancy [126, 127].

The immunogenic dormancy in cancer is maintained by the innate (e.g. NK cells) and acquired (e.g. T cells) immune system, whose interplay is orchestrated by T helper cells [128]. NK cells and tissue-resident CD8 + T cells prevent outgrowth of metastases in murine and human melanoma models (Fig. 2B) [129–131]. The effect is postulated to be rather cytostatic than cytotoxic [61]. In addition, NK cells, CD8 + T cells, and T helper cells secrete cytokines like IFNγ, tumor necrosis factor (TNF), and IL-2, preventing tumor growth and angiogenesis in a melanoma and a pancreatic cancer mouse model (Fig. 2B) [132–135]. Assuming cellular dormancy, single tumor cells must protect themselves from immunogenic eradication for long-term persistence. On the one hand, melanoma cells may bypass the immune system and evade killing by down-regulating major histocompatibility complex I (MHC-I), driven by TGF-β [136]. On the other hand, higher MHC II expression allows binding to lymphocyte activation gene-3 (LAG3) on melanoma-associated T cells, ultimately ensuring melanoma cell survival in vitro (Fig. 2B) [137]. T cells can maintain cancer cells in permanent dormancy and are able to prevent disseminated melanoma cells in visceral organs from expanding [46, 61, 138, 139]. In a mouse melanoma model, intratumoral injection of IL-2 results in decreased tumor growth and long-term tumor dormancy depending on the presence of T cells or natural killer cells [134].

The immune system is also involved in the reawakening of melanoma. Under immunosuppression, for example after organ transplantation, metastases of malignant may occur in patients due to lack of immune control [140]. Furthermore, activation of the immune system, e.g. under systemic inflammation or infiltration of stromal inflammatory cells into the tumor (macrophages, lymphocytes), is associated with lymphatic invasion and cancer recurrence in melanoma and other cancer types (Fig. 2B) [141–143].

ECM and associated factors form the tumor niche, and directly interact with tumor cells. ECM components, like collagen XIV or other fibrillar collagens, display pro-dormancy properties in an in vitro melanoma model [96]. Fibronectin, secreted by melanoma cells themselves, is associated with enhanced migratory and proliferative properties in in vitro assays, as well as expression of EMT markers along with a downregulation of MITF in patient samples (Fig. 2C) [144, 145]. The configuration of ECM components is crucial for its proliferation-promoting or -inhibiting effect. Fibroblast-derived MMP-14 degrades collagen XIV in vitro, therefore antagonizing the growth-arresting effect on melanoma cells [98]. As another variable, the stiffness of a fibrin matrix can be increased. Thus, p21 and p27 are epigenetically upregulated, and dormancy is induced in human and murine melanoma cells under in vitro and in vivo conditions (Fig. 2C) [146].

Last, factors can be bound to ECM, such as proliferation-promoting insulin-like growth factor (IGF) -1 which was proven to enhance proliferation in a 3D melanoma model [147]. Additionally, WNT5A has been identified as activator of dormancy. However, fibroblasts undergo age-related reprogramming and release a soluble antagonist (sFRP1) of WNT5A, thus enabling metastatic outgrowth of melanoma metastases in mice (Fig. 2C) [148].

Some of the above factors may be preferentially assigned to tumor mass, others to cellular dormancy. Nevertheless, a role at both levels cannot be excluded. Analysis at the single cell level could improve our understanding of cellular dormancy induction.

## Neutrophils in melanoma

Macrophages, NK cells and neutrophils account for more than 80% of tumor-associated immune cells in melanoma, of which neutrophil invasion is related to poor prognosis [149–151]. Clinical studies indicated that elevated numbers of circulating neutrophils are an independent marker of adverse prognosis in melanoma patients and negatively predict response towards immunotherapy [152–155]. Upon ultraviolet radiation of melanoma in mice, neutrophils invade into the tumor and induce a more migratory phenotype, local angiogenesis and angiotropism [91]. Thus, local and systemic inflammation by neutrophils may be drivers of melanoma progression. Systemic inflammation may be triggered by smoking or obesity, resulting in increased activation of neutrophils in patients [156–158].

**Fig. 3:**
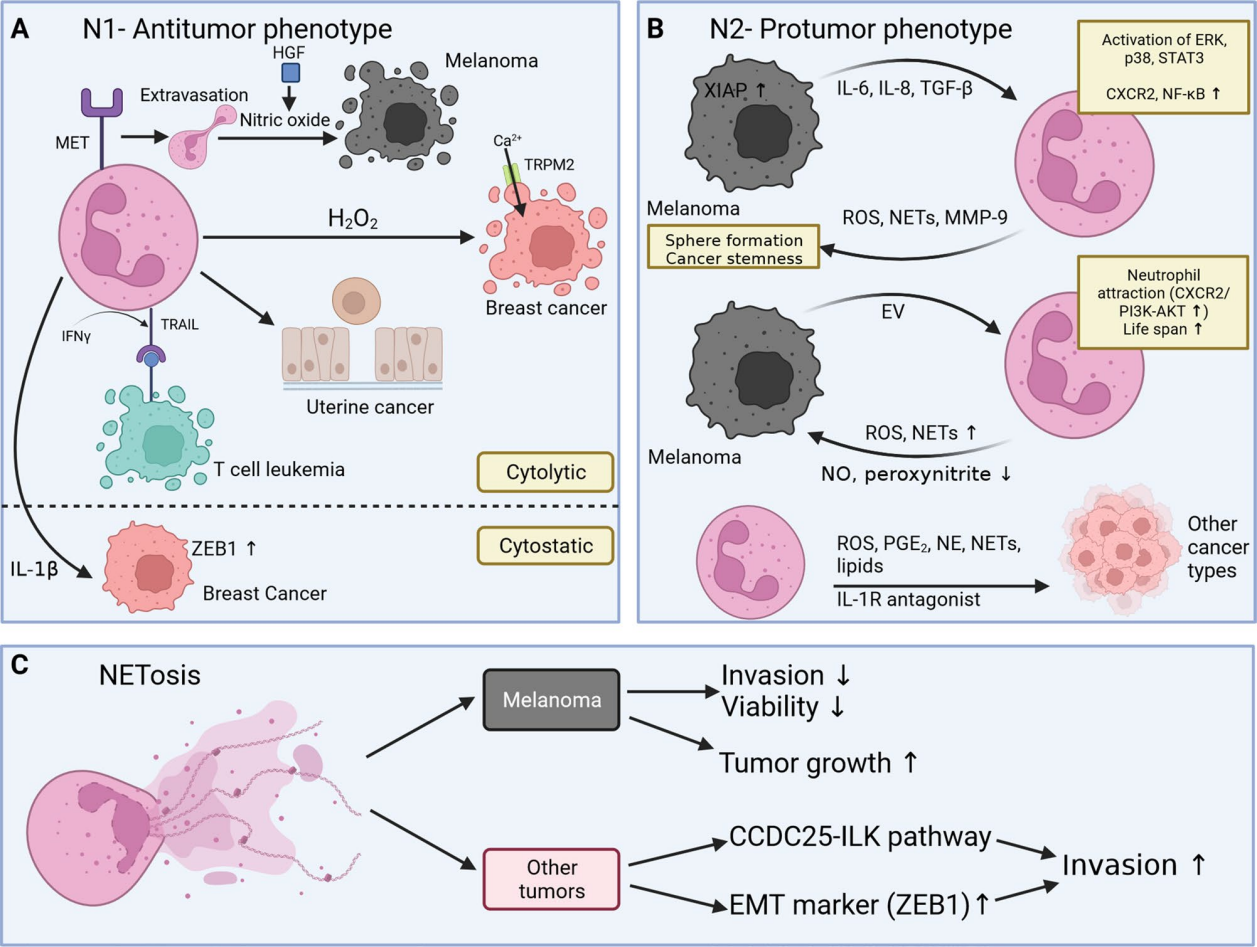
Neutrophils and NETs in cancer. **A** Neutrophils exert their effect in a cytolytic way. Neutrophils extravasate upon MET receptor upregulation, and HGF triggers nitric oxide release to induce cell death in melanoma cells. Hydrogen peroxide (H_2_O_2_) allows cytotoxic Ca^2+^ influx via TRPM2 into breast cancer cells. Neutrophils promote detachment of the basement membrane to make oxygen inaccessible in uterine cancer cells. IFNγ upregulates TRAIL in neutrophils to induce apoptosis in leukemic T cells. IL-1β by neutrophils induces upregulation of ZEB1 (EMT marker) to induce a phenotype switch. **B** Tumor cells may release cytokines or extracellular vesicles (EV) to activate neutrophils. In turn, effectors such as ROS or NETs are released to promote tumor growth. **C** Evidence in melanoma supports a proliferation-promoting role of NETs, impeding invasiveness and cell viability. Other tumor types suggest an invasive phenotype upon NET release (created with BioRender.com)

Neutrophils are a heterogeneous cell population with high plasticity. In melanoma patients, single-cell cytometry by time of flight (CyTOF) identified seven subtypes exhibiting diverse capacities for reactive oxygen species (ROS) release and phagocytosis. Compared to healthy individuals, melanoma patients have more immature neutrophils, which may show a different response towards tumor cells [159]. In the following section, we differentiate only into antitumor (N1) and protumor (N2) phenotypes, with a clear preponderance of evidence on the tumor-promoting side (Fig. 3, A + B) [160].

Antitumor effects include direct cytotoxicity of neutrophils, therefore sustaining a tumor mass dormancy (Fig. 3A) [161]. A melanoma and lung cancer mouse model were able to prove that upregulation of receptor tyrosine kinase MET in neutrophils allows enhanced tumor infiltration. Upon binding of hepatocyte growth factor (HGF), nitric oxide is released to kill melanoma cells [162]. Further antitumoral evidence is limited to other cancer types. ROS (hydrogen peroxide, H_2_O_2_) mediate their cytotoxic effect in breast cancer cells in vitro and in vivo via H_2_O_2_-sensitive TRPM2 channels, resulting in Ca2 + influx [163]. Apoptosis is further induced by IFNγ-dependent stimulation of neutrophils with subsequent expression of tumor necrosis factor-related apoptosis-inducing ligand (TRAIL) towards leukemic T cells in vitro [164]. Neutrophils interfere in tumor-ECM interaction of uterine cancer in mice, leading to detachment from the basement membrane and hypoxic cell death [164, 165]. Neutrophils also harbor cytostatic-inducing properties. Cells of the innate immune system release IL-1β, which upregulates ZEB1 in breast cancer cells and induces growth arrest by EMT [166]. Neutrophils may incorporate other immune cells into tumor suppression, as reviewed elsewhere [160].

Melanoma cells may force neutrophils into an N2-protumoral phenotype (Fig. 3B). This is shown in two recently published melanoma studies [167, 168]. The in vitro model, conducted by Anselmi et al., demonstrates that conditioned medium (CM) of A375 melanoma cancer stem cells (CSC) activates neutrophils. In turn, activated neutrophils favor cancer stemness (ABCG2 upregulation) and sphere formation of A375 cells. In detail, proinflammatory cytokines of the CM (IL-6, IL-8, TGF-β) trigger the activation of ERK, p38 and STAT3 and overexpression of CXCR2 (chemokine receptor of IL-8) and NF-κB in neutrophils. Ultimately, ROS, NETs, MMP-9, and other proinflammatory cytokines are released, making N2 polarization likely [167].

Similarly, extracellular vesicles (EV) derived from MV3 melanoma cells, but not from melanocytes, increase the expression of N2 molecular markers, such as Arg1, CXCR4 and VEGF in neutrophils. Melanoma-derived EVs are able to attract neutrophils by activation of the CXCR2/PI3K-AKT signaling pathway, prolong their survival, and enhance NET release. Additionally, ROS production is increased, which has been previously associated with tumor progression [168]. However, NO and peroxynitrite were stated to be cytotoxic to melanoma cells, and their level is, therefore, reduced in a N2 state of neutrophils. In contrast, LPS increases NO and peroxynitrite levels and increases neutrophil toxicity toward melanoma cells, implicating a stimulus-dependent differentiation of PMN [169].

X-linked inhibitor of apoptosis (XIAP) has previously been linked to melanoma progression by its anti-apoptotic function in cancer. However, emerging in vitro and in vivo evidence in attributes XIAP a role in neutrophil attraction through IL-8 and subsequent melanoma progression [170].

The pro-tumoral activity of neutrophils has been observed in other tumor types as well. ROS oxidatively damages DNA, thus promoting invasiveness in intestinal cancer of mice [171]. Proinflammatory signals, like prostaglandin E_2_, NE and mitochondrial-dependent NET, released from neutrophils promote proliferation in lung and anaplastic thyroid cancer [172–174] while IL-1 receptor antagonist blocks the transition to senescence in prostate cancer in vitro and in vivo [175]. Again, neutrophils may exert their protumor effects by interacting with other immune cells or the TME [160].

In summary, evidence for the tumor-maintaining and growth-promoting effects of neutrophils predominates in melanoma as well as in other tumor cells.

## NETs in melanoma

Neutrophils shape the TME by release of neutrophil extracellular traps (NETs), a process that has recently been studied with great interest [176]. NETs are large extracellular DNA-protein web-like structures, which are known to be released by neutrophils in response to infection and kill microbes by trapping [177]. NETs themselves create a proinflammatory environment and recruit additional neutrophils, which in turn can form new NETs – this constitutes a vicious circle that ultimately promotes angiogenesis and tumor growth [151, 178]. Interestingly, NETs can be induced in neutrophils not only by an infectious stimulus, but also by metastatic (and not by non-metastatic) cancer cells [151, 156].

Knowledge of NETs in melanoma is limited and controversial. Schedel at al [179]. show that NETs are found in ulcerated melanoma, but their quantity does not correlate with the patient’s prognosis. Melanoma cells bind integrin-mediated to NETs and are hindered in their invasion into Matrigel in vitro. NETs are applied at doses ranging from 5 to 93 ng/µl which results in reduced cell viability in a dose-dependent manner (Fig. 3C). This effect is reversible by DNase I and cannot be induced by genomic neutrophil DNA. In summary, NETs show an antitumoral effect in vitro. These findings are confirmed in an in vivo model of melanoma, in which protumoral, IFNβ- deficient neutrophils only provide low levels of NETs and insufficient tumor control [180]. Interestingly, the opposite effect was shown in a melanoma mouse model (Fig. 3C). Neutrophils are primed towards NETosis by granulocyte colony stimulating factor (G-CSF) and promote tumor growth [178.] The previously described studies also suggest that NET release is associated with melanoma progression.

Possible mechanisms of action of NETs in cancer have been reported in other tumor types [181–183]. In this context, the direct effect of NETs on cancer cells seems to promote invasiveness. In breast cancer cells, NETs have been shown to bind to the CCDC25 receptor, thereby increasing cell motility in mice via the integrin-linked kinase (ILK) pathway [181]. In addition, breast and colorectal cancer cells transform into an invasive, mesenchymal (ZEB1-positive) state upon NET exposure (Fig. 3C) [182, 183].

## The role of neutrophils and NETs in melanoma dormancy

Few studies address the effect on NETs in dormant cancer cells. The different pro- and antitumor effects do not allow clear conclusions whether NETs induce reawakening or sustain a dormant state. NETs mediate their effects partly via ECM components, and in vivo also via other immune cells or angiogenesis. This must be considered as a limitation of in vitro studies. Ultimately, the effect by NETs may also depend on the cell line studied. The mechanism behind the neutrophil-mediated enhancement of tumor progression has been partly unraveled in breast and prostate cancer models.

Interestingly, it has been shown in breast cancer patients and mouse models that circulating tumor cells form clusters with neutrophils in the blood releasing cytokines such as IL-6 or G-CSF, which drives tumor proliferation and increases their metastatic potential [184]. These cytokines are also released during chemotherapy of breast cancer cells with taxanes through stromal damage and increased neutrophil infiltration and lead to reawakening of dormant cancer cells in in vitro and in vivo models [185]. Surgical stress is able to elevate levels of IL-6 and IL-8 in a murine breast cancer model, which could in turn stimulate neutrophils [186]. It was recently shown in an in vivo breast cancer model that - depending on the spectrum of chemoattractants in the tumor microenvironment (e.g. CXCL1 or S100A8)- N1- or N2-polarized neutrophils are attracted and exert different functions, whereby N1- antitumor neutrophils can maintain dormancy [187]. In addition, stress- activated neutrophils lead to dormancy escape in lung and ovarian cancers by release of oxidized lipids [188].

In breast cancer, integrin and extracellular matrix remodeling events have previously been described as central dormant cell reactivation mechanisms [38, 189]. Interestingly, it was shown that cancer cells can hijack neutrophils to increase metastatic spread through the induction of NETs [156]. NETs initiate awakening of dormant breast cancer cells in vivo by remodeling the ECM in the dormant niche leading to FAK/ERK/MLCK/YAP signaling in cancer cells. Toll-like receptor 9 (TLR9) in colon cancer cells binds extracellular DNA in vitro and is capable to promote tumor survival and progression through induction of autophagy [190]. NETs are also able to promote endothelial-mesenchymal transition through the subsequent activation of β-catenin signaling in vitro [190, 191]. Therefore, current evidence in several tumor models in vitro and in vivo suggests that neutrophils and NETs lead to cancer recurrence, which is in part mediated by ECM remodeling. Nevertheless, not many data are available for melanoma in this respect and therefore further studies should be conducted in suitable in vitro and in vivo melanoma models.

NET signaling seems to be different from signaling by extracellular DNA released from dying cells. It has been shown that extracellular DNA can signal through DNA receptors such as RAGE or some cytosolic nucleic acid sensors initiating activation of the cGAS–cGAMP–STING pathway [192, 193]. cGAS is a cytosolic DNA sensor that activates innate immunity by initiating the STING-IRF3-type I IFN signaling cascade. Interestingly, activation of this pathway promotes cellular senescence and is not involved in reawakening of dormant cancer cells [194–196]. Therefore, the mechanistic details of the role of immune cells, NETs or extracellular DNA in the niche in fostering dormancy or reawakening dormant tumor cells remain unknown.

## In vitro and in vivo dormancy models in malignant melanoma

Dormancy models range from 2D and 3D in vitro models, to mouse and zebrafish in vivo models. The main challenges of the models include the long latency of clinical dormancy and the complex interplay of the immune system [197].

2D in vitro models allow well-controlled conditions and are suitable for high throughput screening. As reported by Pradhan et al., dormancy can be induced by ECM, cell signaling (cytokines), biochemically (e.g. hypoxia) or by chemotherapy [198]. Accordingly, a rigid fibrin matrix induces growth arrest in a 2D melanoma model [146]. In a recently published in vitro model, senescence was induced by cytokines (IFNγ, TNF), as well as by 24–96 h treatment with chemotherapeutic agents (palbociclib, doxorubicin). Stable growth arrest was induced for one week, however, its validity for long-term dormancy is limited [199]. This is clearly demonstrated in a study of breast and prostate cancer: after incubation with doxorubicin or docetaxel for 2–4 days, reproliferation was not apparent until day 18–22 after treatment [200]. Ultimately, in vitro models of melanoma may be used to elucidate molecular mechanisms of dormancy induction and reawakening at the bulk or single cell level [100, 112].

More complex 3D in vitro models such as spheroids or organoids mimic cell-matrix and cell-cell interaction in melanoma. Compared to monolayer culture, the conditions in spheroids result in a dormant, invasive phenotype, possibly triggered by surrounding ECM, hypoxic conditions or close intercellular contact [201, 202]. Adding cellular components, such as fibroblasts and immune cells, to organoids provides evidence of the interplay between immune cells and ECM. Exemplary, loss of HAPLN1 (link protein of proteoglycans and hyaluron) release by fibroblasts resulted in an aligned ECM structure and elevation of the metastatic potential in melanoma. In contrast, mobility of T cells and polymorphonuclear cells was inhibited, therefore changing the immunogenic microenvironment [203].

In vitro dormancy models for breast cancer have been recently summarized [204]. Until now only few in vitro and also in vivo models investigate tumor dormancy in melanoma, and these were discussed already in this review, some of which provide limited evidence due to short follow-up [146, 199, 205]. In vivo tumor dormancy models have been recently reviewed extensively by Mahmoud et al. [206] and Gu et al. [207] and with a focus on melanoma in vivo models by Patton et al. [197] and will therefore not be discussed in detail here. Unfortunately, not many in vivo studies have been published until now concerning melanoma dormancy, some of those were already described in this review. In a syngeneic mouse model of melanoma dormancy and GFP-labelled dormant cell-derived cell lines it was shown that vaccination against melanoma did not prevent tumor cell dissemination and induced dormancy in vivo, which was regulated by glucocorticoid-induced leucine zipper (GILZ)-mediated immunosuppression [31]. To follow the dynamics of melanoma cell dissemination, niche occupancy, dormancy induction and reactivation the interaction between cancer cells and the microenvironment has to be studied in real time, e.g. by live cell imaging. High-resolution imaging of fluorescently labeled melanoma cells has been used in a zebrafish model [208]. It has been shown that after metastatic dissemination, keratinocyte-derived endothelin 3 (EDN3) the microenvironment provides signals to promote phenotype switching between invasive and proliferative states and provide proof that targeting tumor cell plasticity is a valuable therapeutic option. Using lineage tracing in a spontaneous metastatic melanoma mouse model it was shown that disseminated quiescent melanoma cells reside in intravascular niches in metastatic organs and are able to transdifferentiate into endothelial cells [59]. The ‘‘MET Alert’’ mouse, engineered with a VEGFR3-luciferase reporter expressed specifically in lymphatic vessels reveals distinct patterns of metastatic progression of melanoma [209].

A great help in the visualization of the induction of tumor dormancy and reactivation in vitro or in vivo is the use of fluorescence reporter systems such as the fluorescence ubiquitination-based cell cycle indicator (FUCCI) system. In this construct, monomeric Azami Green (mAG) is bound to hGEM (geminin), while monomeric red fluorescent dye Kusabira Orange 2 (mKO2) is bound to the hCDT1 [210]. As these proteins are ubiquitylated by different E3 ligases, APC^Cdh1^ and SCF^Skp2^ respectively, which are expressed in a cell cycle-dependent manner the cells end up changing their fluorescent signal accordingly. While a red signal can be detected in G1 phase, a green signal is visible in G2/M phase. Meanwhile, the transition from G1 to S phase results in an expression of both, which results in a yellow signal. It is also possible for the FUCCI transfected cells to temporarily show no fluorescence at all, while in between phases. Using live cell imaging of the stably transfected cells the real-time cell cycle stage cells resided in can be detected and estimate cell proliferation over time. Another interesting method to track single tumor cells in heterogeneous cell populations in mice is the novel methodology CaTCH (CRISPRa tracing of clones in heterogeneous cell populations) which enables lineage tracing of a barcoded small cell population [211, 212].

## Therapeutic implications

The knowledge cited above and the ever-increasing insights into the molecular and immunological mechanisms of the induction of tumor dormancy and their reactivation is important to design novel therapies to cure cancer patients or prolong their life. To achieve this, several problems have to be solved. First, the identification of gene signatures or biomarkers indicative for the existence of dormant cancer cells in patients are necessary. The use of single cell or spatial transcriptomics would greatly help in this respect. Second, sophisticated 3D human modular in vitro and in vivo tumor dormancy models are needed to evaluate the complex interplay of tumor cells with microenvironmental factors and immune cells. Third, it has to be evaluated in the respective models which of the two different treatment options are more promising: either one keeps tumor cells in a dormant state, which implies the need to carry out life-long treatment or one activates dormant cells in order to improve the efficacy of antiproliferative therapies [65, 114].

It still needs to be investigated which role an inflammatory niche environment has on inducing tumor dormancy or reactivate those cells. Especially, novel immune targeting strategies in clearing dormant cancer cells have to be evaluated. In addition, the role of innate immune cells such as neutrophils, macrophages or myeloid-suppressor cells needs to be investigated in more detail by functional analyses in vitro and in vivo. Dormant tumor cells hiding in niches must be better characterized in patient tissue samples by highly advanced technological platforms (single cell technologies, highly multiplexed microscopy, imaging, bioinformatic analyses and machine learning tools). Based on this knowledge the path is clear to perform clinical studies for test effective therapeutic strategies in patients with dormant cancers [46, 114, 213].

## Conclusions

The molecular and immunological mechanisms involved in tumor cell dormancy and awakening are complex and seem to be different for each tumor type. Therefore, complex 3D human in vitro models, sophisticated in vivo models using live cell imaging and analysis of patient material using single cell and spatial transcriptomics as well as functional assays taking into account the interplay of dormant cancer cells with the tumor microenvironment are necessary to find novel therapies to eliminate dormant cancer cells. Findings of 2D models should be transferred to more complex 3D models, as they allow approximation to more complex in vivo conditions. Since phenotype switching has been shown to promote melanoma metastasis, lineage tracing strategies in combination with single-cell multi-omics methods in suitable melanoma mouse models have still to be performed to follow the temporal fate of single cells from dissemination to metastasis to provide direct evidence of the molecular and phenotypic changes melanoma cells pass in order to metastasize [214]. Furthermore, spatial mapping of the phenotypic cell states in dormant melanoma cells in their niches and their molecular characterization on single cell level will further improve our understanding of the influence of the niche environment on phenotype plasticity and the establishment of persistent melanoma cells. Each therapeutic strategy must consider the intra-tumor heterogeneity and cellular plasticity to prevent phenotype switching towards treatment-resistant subpopulations. The time has come to uncover the secrets of dormant cancers and to design novel effective treatment strategies to eliminate dormant tumor cells to prolong patient’s survival.

## Data Availability

Not applicable.
